# Involvement of Endocytosis and Alternative Splicing in the Formation of the Pathological Process in the Early Stages of Parkinson's Disease

**DOI:** 10.1155/2014/718732

**Published:** 2014-04-03

**Authors:** Anelya Kh. Alieva, Maria I. Shadrina, Elena V. Filatova, Aleksey V. Karabanov, Sergey N. Illarioshkin, Svetlana A. Limborska, Petr A. Slominsky

**Affiliations:** ^1^Institute of Molecular Genetics, Russian Academy of Sciences, Moscow 123182, Russia; ^2^Research Centre of Neurology, Russian Academy of Medical Sciences, Moscow 125367, Russia

## Abstract

Parkinson's disease (PD) is the one of most widespread neurodegenerative pathologies. Because of the impossibility of studying the endogenous processes that occur in the brain of patients with PD in the presymptomatic stage, the mechanisms that trigger the disease remain unknown. Thus, the identification of the processes that play an important role in the early stages of the disease in these patients is extremely difficult. In this context, we performed a whole-transcriptome analysis of the peripheral blood of untreated patients with stage 1 PD (Hoehn-Yahr scale). We demonstrated a significant change in the levels of transcripts included in the large groups of processes associated with the functioning of the immune system and cellular transport. Moreover, a significant change in the splicing of genes involved in cellular-transport processes was shown in our study.

## 1. Introduction

Parkinson's disease (PD) is one of the most widespread neurodegenerative pathologies [1]. A key component of the pathogenesis of PD is the death of nigrostriatal neurons in the midbrain of patients [2], which in turn leads to a decrease in the concentration of dopamine (DA) and a disturbance in signal transmission between brain parts [3].

Genetic mutations that lead to the development of monogenic forms of PD, such as those located in the* SNCA*,* LRRK2*,* PARK2*,* PINK1*,* PARK7*, and* ATP13A2* genes, have been identified. A large number of candidate genes that may also contribute to the development of the pathogenesis of PD have been described [4, 5]. Investigations of the functions of these genes have shown that the disturbance of cell processes related to mitochondrial dysfunction, oxidative stress, proteolysis, and immune response may play an important role in the pathogenesis of PD. Despite the many years dedicated to identifying the molecular-genetic factors that underlie the development of the PD pathogenesis, the full picture of the etiopathogenesis of PD has not been elucidated. Accordingly, the mutations that are known currently as being causative of monogenic forms of PD are only responsible for about 5–10% of all cases of familial PD [6]. Therefore, it is necessary to continue searching for new genes that are associated with the development of the pathological process in PD. One of the potential approaches that can be used to address this problem is the study of transcriptome changes in PD. To date, a large number of studies of the transcriptome profile of the brains of patients with PD have been performed. However, the patients who were analyzed in those reports were in the final and most severe stages of the disorder and underwent active medical treatments [7–10]; therefore, the data on gene-expression changes obtained in those studies do not represent the processes of initiation of the development of the disorder.

Because of the impossibility of studying the endogenous processes that occur in the brain of patients with PD in the presymptomatic stage, the mechanisms that trigger the disease remain unknown. To date, several studies of transcriptome changes in the peripheral blood of patients with PD have been reported [11–13]. Although the results of those studies are of great interest, the patients analyzed by those authors were in the progressive stages of PD and were under active drug treatment. In this context, we performed a whole-transcriptome analysis of the peripheral blood of untreated patients with stage 1 PD (Hoehn-Yahr scale).

## 2. Materials and Methods

### 2.1. Patients

All patients (Slavs residing in the European part of Russia) were diagnosed with PD at the Research Center of Neurology, Russian Academy of Medical Sciences. All patients with PD were selected and studied according to the International Unified Parkinson's Disease Rating Scale (UPDRS) and Hoehn-Yahr scores [14, 15]. The diagnosis of PD was based on the UK PD Brain Bank Criteria [16]. In this work, four untreated patients with stage 1 PD were studied. The mean age ± SD at the disease onset was 55.0 ± 5.0 years (range: 50–60 years), and the mean age at enrollment was 55.0 ± 5.0 years (range: 50–60 years). Four neurologically normal age-matched individuals from the same population were studied as controls.

All participants were examined using the MLPA procedure [17, 18], which revealed an absence of mutations. All blood samples were collected with the informed consent of the investigated persons. The study was approved by The Ethics Committee of the Research Centre of Neurology, RAMS.

### 2.2. RNA Preparation

All blood samples were collected at 8:00 a.m. while fasting and then stored for less than 2 h at +4°C before isolation of RNA. The isolation of total RNA from whole blood was performed using the ZR Whole-Blood Total RNA Kit (Zymo Research Corp., Irvine, CA, USA) according to the manufacturer's recommendations. RNA concentration was determined using the fluorometric Qubit 1.0 by Quant-iT RNA BR Assay Kit (Life Technologies, Carlsbad, California, USA).

### 2.3. Whole-Transcriptome Analysis

The analysis of large-scale transcriptome changes was carried out both in individual pairs (PD patient/healthy control) and in RNA pools (pool of the RNAs from all patients with PD/pool of the RNAs of healthy controls). Two hundred nanograms of total RNA derived from each sample of the peripheral blood of patients or controls was included in each corresponding pool. Hybridization was performed using the HumanHT-12 v4 Expression BeadChip Kit (Illumina, San Diego, California, USA). Ten independent hybridizations were carried out, and the expression levels of all genes were determined for each “sample/control” pair and for pool pairs.

The data obtained were compared among pairs and pools. Averaged data regarding transcript levels in pools and pairs were compared with the averaged data in the other pairs and pools using the Genome Studio software (Illumina, San Diego, California, USA). Our data are available as gene expression omnibus (GEO) datasets (http://www.ncbi.nlm.nih.gov/geo/query/acc.cgi?acc=GSE54536) [19, 20].

### 2.4. Statistical Analysis

A volcano plot was used to identify differentially expressed genes (using *n*-fold ≥1.5 and *P* value <0.01 as the threshold of statistical significance). For further investigations, we selected transcripts that were common among all the pairs and pools. The software package Genome Studio (Illumina, San Diego, California, USA) and the DAVID Bioinformatics Resources 6.7 database (http://david.abcc.ncifcrf.gov/home.jsp) were used for the statistical processing of the data regarding the gene-expression levels obtained from microchips [21, 22].

### 2.5. Analysis of Obtained Data

We carried out a transcriptome analysis in samples of the peripheral blood of four untreated patients with stage 1 PD, according to the Hoehn-Yahr scale [15], and four healthy volunteers using the HumanHT-12 v4 Expression BeadChip Kit. The relative levels of 47,000 transcripts were evaluated using this chip. In a first stage, five pairs of total RNA samples were used for whole-transcriptome analysis: four pairs that each consisted of the RNA from a patient with PD and the RNA from a healthy volunteer with matching age and sex and one pool pair that consisted of a pool of the total RNA from all patients investigated and a pool of the total RNA from the healthy controls. First, differentially represented transcripts (DRT), that is, those with levels that were altered by more than 1.5 times (*P* value <0.01), were selected for each pair. Subsequently, the pairs were compared with each other, and the DRTs that were common to all five pairs were taken into consideration for further analysis (Table 1). In total, 1429 DRTs were selected as a result of the analysis performed using the Genome Studio software (Illumina, San Diego, California, USA) (*n*-fold ≥ 1.5 and *P* value <0.01). The removal of individual differences between pairs yielded the most probable and significant results.

A cluster analysis of these DRTs was performed using the DAVID Bioinformatics Resources 6.7 database [21, 22]. DAVID was used for further analysis of a panel of differentially expressing genes, which allowed us to perform a fast annotation of genes of interest and to combine them into functional groups. Two hundred eighty-eight transcripts for which there were no descriptions in the available databases were excluded from further analysis. Processes showing statistical significance, as evaluated based on the indicators of an enrichment score > 1.0, *P* value  < 0.01, and FDR < 0.05, were selected from the total amount of data during the cluster analysis.

It is worth mentioning that the calculation of the false-discovery rate (FDR) or the correction of values using the Benjamini-Hochberg method is applied in situations where it is necessary to make a common decision on any matter in the presence of information regarding many parameters [23]. Currently, there is no established system of the application of this correction, and results with an FDR < 0.1 were taken into consideration in some studies [24] whereas an FDR < 0.05 was used in other studies [25]. In our work, we adhered to the more stringent version of this correction and took into account values with an FDR < 0.05.

## 3. Results and Discussion

The results of the functional clustering of differentially expressed genes are presented in Table 2. Table 2 shows summarized data after clustering by DAVID. Data on the expression changes of the individual genes are available as GEO datasets (http://www.ncbi.nlm.nih.gov/geo/query/acc.cgi?acc=GSE54536).

The data presented in Table 2 revealed that two clusters of metabolic processes associated with the functioning of the immune system and the processes of cellular transport and a cluster of genes actively undergoing alternative splicing were identified as a result of a bioinformatics analysis of DRTs. The high statistical significance of the clusters detected suggests that the biological processes included in these clusters may play an important role in the initiation of the pathological process in the early stages of PD.

At present, it is known that chronic inflammation is a characteristic of many neurodegenerative diseases [26] including PD. Moreover, the activation of microglia in the midbrain is positively correlated with the manifestation of the motor symptoms of PD [27, 28]. Experiments performed in vitro showed that misfolded synuclein activates microglia, which in turn leads to the secretion of cytokines, such as TNF-*α* [29] and IL-1*β* [30], and to the production of ROS, which damage DA neurons [31]. After several GWASs and meta-analyses of the data obtained in these studies, the association between loci located within the gene clusters of histocompatibility (*HLA-DRA* and* HLA-DRB*) and PD was demonstrated [32, 33]. We identified four processes related to the immune system in our work, with a high degree of significance (*P* value <1.3*E* − 5 and FDR < 5.5*E* − 3): immune system process, defense response, response to cytokine stimulus, and positive regulation of I*κ*B kinase/NF-*κ*B signaling. The statistical significance of the results of the functional-validation analysis suggests that the processes associated with the functioning of the immune system may play an important role in the development of PD. Furthermore, a change in the expression pattern of genes associated with histocompatibility gene clusters, such as* HLA-A*,* HLA-C*,* HLA-DRB4*,* HLA-DRB1*,* HLA-DRB6*,* HLA-DQA1*, and* HLA-DQB1*, also indicates a similar role for these genes. Concomitantly, the* HLA-DRB4*,* HLA-DRB1*, and* HLA-DRB6* genes are located in the genomic region that was shown to be associated with PD in GWASs [32, 33].

Another group of processes that yielded results with a high degree of significance includes those related to transport (Table 2). Data accumulated in numerous studies suggest that abnormalities related to the functioning of vesicular transport play an important role in neurodegeneration. Thus, disturbance of vesicular transport and, consequently, synaptic transmission is a common feature of diseases such as PD, Alzheimer's disease, and several other disorders, although abnormalities at the synapse either precede or accompany the onset of symptoms [34–38]. The processes associated with the functioning of the endosome (*P* value = 2.8*E* − 6 and FDR = 3.6*E* − 4) and vesicular transport (*P* value = 3.1*E* − 6, FDR = 2.0*E* − 3) exhibited the highest reliability in this group. These data also indicate that abnormalities of vesicular transport may be involved in PD.

The third cluster that drew our attention was a cluster of genes with alternative splicing, which exhibited the highest statistical significance (*P* value = 1.9*E* − 10 and FDR = 5.0*E* − 8). This cluster included mainly genes actively undergoing alternative splicing. Currently, it is known that alternative splicing may affect processes that are directly related to the functioning of the nervous system, such as synapse formation [39] and migration of nerve cells [40]. According to some studies that were performed using the brains of humans and chimpanzees, the intensity of the alternative splicing varies according to age [41]. In addition, relationships between changes in the intensity of alternative splicing and neurodegenerative diseases, such as Alzheimer's disease [42] and amyotrophic lateral sclerosis [43], were demonstrated.

Therefore, we decided to examine in greater detail the genes included in the cluster of alternative splicing; reclustering of this group of genes was performed using DAVID. The results listed in Table 3 revealed that alternative splicing occurred mainly in genes that are involved in the functioning of the cellular transport.

As can be seen from Table 3, genes, involved in the functioning of cellular transport, are mainly alternatively spliced.

It should be noted that, for the majority of the genes, a decrease in the level of their respective mRNAs compared with the control was observed. Concomitantly, the chip allows us to analyze only the level of the basic transcripts of most of the genes. Thus, the decrease in mRNA levels observed in our work may be associated with the intensification of alternative splicing in the peripheral blood of patients, leading to a reduction of the primary transcript. This points indirectly at elevated levels of alternative transcripts and, consequently, at the accumulation of proteins with altered functions. For example, a 1.5-fold decrease in the level of the primary transcript of the* SNCA* gene compared with the control was found here. These data may indicate an intensification of the alternative splicing of* SNCA*, which may lead to the accumulation of alternative transcripts and an increased synthesis of protein entities with a modified structure. It is known that an increased proportion of alternatively spliced transcripts lacking either exon 4, exon 6, or both leads to the formation of unstable heterotetramers that dissociate easily, thus resulting in the accumulation of toxic oligomers [44].

Moreover, genes that are directly involved in vesicle-mediated transport, such as dynamin 2 (*DNM2*), adaptor-related protein complex 2 (*AP2*), syntaxin-2 (*STX2*), syntaxin-10 (*STX10*), VAMP-associated protein A (*VAPA*), vesicle-associated membrane protein 4 (*VAMP4*), and* VAMP8*, were also included in this cluster. The level of their respective transcripts in the peripheral blood of patients with PD was reduced, on average, by 1.5 times compared with the control. This indicates that there is a change in the intensity of the transport of synaptic vesicles in PD. In addition, a reduction of endocytosis activity was observed. This type of effect has been observed in model organisms with induced PD [45]; however, our data were generated using samples of the peripheral blood of patients with PD, which represents the first report of this kind.

## 4. Conclusion

In this study, we demonstrated a significant change in the levels of transcripts included in the large groups of processes associated with the functioning of the immune system and cellular transport. Moreover, a significant change in the splicing of genes involved in cellular-transport processes was shown in our study. Alternative splicing should be considered as another pathway of regulation of gene expression [46]. In most cases, changes in the alternative splicing of genes lead to a decrease in the levels of basic transcripts and are likely to increase the levels of alternative transcripts. It is possible that disturbances in the functioning of the vesicular transport are associated with changes in the alternative splicing of genes that encode proteins that are directly involved in endocytosis and exocytosis. In general, it seems that several independent events that occur in nerve cells, such as the disturbance of processes of vesicular transport and of the immune system and possibly even several currently unknown processes, affect the development of PD simultaneously.

## Figures and Tables

**Table 1:** The amount of DRTs in the pairs investigated (*n*-fold > 1.5 and *P* value < 0.01).

Pair	Amount of DRTs (*n*-fold > 1.5 and *P* value < 0.01)	Amount of common DRTs
1	2676	1429
2	2458	
3	2774	
4	2667	
Pool	1993	

**Table 2:** Results of cluster analysis of 1429 transcripts by DAVID.

Cluster	Gene ontology terms and annotations	Count*	Enrichment score**	*P* value	FDR***
Processes related to functioning of immune system					
Immune system process	GO:0002376	100	6.21	1.8*E* − 7	5.4*E* − 4
Defense response	GO:0006952	68	3.72	8.0*E* − 7	6.2*E* − 4
Response to cytokine stimulus	GO:0034097	17	2.92	1.2*E* − 5	5.5*E* − 3
Positive regulation of I-kappaB kinase/NF-kappaB signaling	GO:0043123	19	2.82	1.3*E* − 5	5.1*E* − 3

Processes related to cellular transport					
Endosome	GO:0005768	41	4.12	2.8*E* − 6	3.6*E* − 4
Vesicle-mediated transport	GO:0016192	63	3.52	3.1*E* − 6	2.0*E* − 3
Protein kinase cascade (intracellular signal transduction)	GO:0035556	48	3.04	5.6*E* − 7	8.8*E* − 4
Membrane-enclosed lumen	GO:0031974	141	2.3	6.4*E* − 4	2.0*E* − 2
Intracellular transport	GO:0046907	66	1.55	2.9*E* − 5	8.1*E* − 3
Endoplasmic reticulum lumen	GO:0005788	14	1.50	6.7*E* − 4	1.9*E* − 2

Other processes					
Alternative splicing	GO:0008380	514	9.31	1.9*E* − 10	5.0*E* − 8
Lysosome	GO:0005764	21	2.64	2.1*E* − 4	1.4*E* − 2
Regulation of catalytic activity	GO:0050790	77	2.23	1.5*E* − 4	2.9*E* − 2
Regulation of molecular function	GO:0065009	85	2.23	2.3*E* − 4	3.9*E* − 2
Regulation of catalytic activity	GO:0050790	77	1.49	1.5*E* − 4	2.9*E* − 2

*Count is number of genes in a cluster.

**Enrichment score ranks the biological significance of gene groups based on overall EASE scores of all enriched annotation terms.

***False-discovery rate (FDR) or the correction of values using the Benjamini-Hochberg method.

**Table 3:** Results of functional reclustering of transcripts from cluster of splicing by DAVID.

Clusters	Pathways	Enrichment score	Count	*P* value	FDR
Membrane-bounded organelle	GO:0043227	4.08	260	1.8*E* − 3	5.0*E* − 3
Endosome	GO:0005768	2.28	20	1.8*E* − 3	4.7*E* − 2
Microtubule cytoskeleton	GO:0044422	2.27	31	5.3*E* − 4	2.4*E* − 2
